# Evaluation of different versions of the gene encoding green fluorescent protein (GFP) in plants

**DOI:** 10.1186/1753-6561-5-S7-P183

**Published:** 2011-09-13

**Authors:** Camila B Scalco, Rochele P Kirch, Giancarlo Pasquali

**Affiliations:** 1 Biotechnology Center, Federal University of Rio Grande do Sul, Porto Alegre, RS, Brazil

## 

The main purpose of the “CDA *Eucalyptus*: Collaborating Center in Agriculture Defense Relative to the Biosafety of Genetically Modified (GM) Eucalypts” is to gather, assess and validate the existing information concerning GM and non-GM *Eucalyptus* and its derivatives in the Brazilian environment, both in laboratories and field test experiments. Accordingly, the purpose of the present research activity is to provide a binary plasmid collection containing different versions of the gene encoding green fluorescent protein (GFP), originally from *Aequorea victorea*, for the future generation of easily detectable GM plant phenotypes. Using commercially available plasmids encoding blue (ECFP) and yellow (EYFP) versions of GFP for expression in bacteria, the initial strategy was to transfer the coding sequences to the intermediate plasmid pSport1 (Invitrogen) and, subsequently, to pART7 plasmid, which contains the promoter and terminator sequences for gene expression in plants. Although recombinant bacteria have been obtained for both genes, DNA sequencing showed that success was achieved only with pSport1-*eyfp*. So far, pART7 versions were not obtained. A new cloning strategy was proposed, which involves the binary plasmid pCAMBIA1302 (Cambia). This plasmid already contains one version of the *gfp* gene for expression in plants. The intention is to replace the *gfp* gene by *ecfp*, *eyfp* and *mCherryFP* versions. As soon as plasmids are finished, plants will be transformed via *Agrobacterium tumefaciens*, and their transgenic state will be confirmed by the fluorescence of the encoded proteins. Thus, the fluorescence of the GFP different versions will be used to monitor seeds, pollen, leaves and transgenic plants as a whole in the environment.

Financial support: Brazilian Ministry of Agriculture, Farming & Supply (MAPA), and The National Council for the Development of Science & Technology (CNPq), Brazilian Ministry of Science & Technology (MCT).

